# Macrophages Inhibit Neovascularization in a Murine Model of Age-Related Macular Degeneration

**DOI:** 10.1371/journal.pmed.0030310

**Published:** 2006-08-15

**Authors:** Rajendra S Apte, Jennifer Richter, John Herndon, Thomas A Ferguson

**Affiliations:** Department of Ophthalmology, Washington University School of Medicine, St. Louis, Missouri, United States of America; Moorfields Eye Hospital, United Kingdom

## Abstract

**Background:**

Age-related macular degeneration (AMD) is the leading cause of blindness in people over 50 y of age in at least three continents. Choroidal neovascularization (CNV) is the process by which abnormal blood vessels develop underneath the retina. CNV develops in 10% of patients with AMD but accounts for up to 90% of the blindness from AMD.

Although the precise etiology of CNV in AMD remains unknown, the macrophage component of the inflammatory response, which has been shown to promote tumor growth and support atherosclerotic plaque formation, is thought to stimulate aberrant angiogenesis in blinding eye diseases. The current theory is that macrophage infiltration promotes the development of neovascularization in CNV.

**Methods and Findings:**

We examined the role of macrophages in a mouse model of CNV. *IL-10* ^−/−^ mice, which have increased inflammation in response to diverse stimuli, have significantly reduced CNV with increased macrophage infiltrates compared to wild type. Prevention of macrophage entry into the eye promoted neovascularization while direct injection of macrophages significantly inhibited CNV. Inhibition by macrophages was mediated by the TNF family death molecule Fas ligand (CD95-ligand).

**Conclusions:**

Immune vascular interactions can be highly complex. Normal macrophage function is critical in controlling pathologic neovascularization in the eye. IL-10 regulates macrophage activity in the eye and is an attractive therapeutic target in order to suppress or inhibit CNV in AMD that can otherwise lead to blindness.

## Abstract 2

Apte and colleagues examined the role of macrophages in a mouse model of choroidal neovascularization, and showed that normal macrophage function is critical in controlling pathologic neovascularization in the eye.

## Editors' Summary

**Background.**

The most common cause of poor eyesight in later life in the developed world is known as age-related macular degeneration (AMD). The macula is the central part of the retina (the film-like membrane at the back of the eye), which is the most sensitive and important for sharp central vision. There are two types of advanced AMD: so-called wet, or neovascular, AMD (neovascular means “new vessel”) and dry, or geographic atrophy, AMD (atrophy means “to waste away”). Wet AMD occurs when abnormal, fragile new blood vessels grow under the macula behind the retina. These blood vessels often leak blood and fluid, which lift the macula. Dry AMD occurs as the light-sensitive cells in the macula (the rods and cones) break down. To study this disease further, researchers use animal models. One such animal model is made by using a laser to damage the back of the eye in a mouse, which causes the formation of new vessels. Various treatments can then be tested to see if they have any effect on the damage.

**Why Was This Study Done?**

One theory about AMD is that the immune system may be involved in determining how severe the damage at the back of the eye is, and how much new vessel formation occurs. The researchers wanted to look at the effect of the immune system on AMD, in particular, the effect of one type of cell called a macrophage, and a substance, IL-10, that is secreted from bone marrow cells and that affects how these macrophages work.

**What Did the Researchers Do and Find?**

The researchers used a mouse strain in which IL-10 was absent, induced damage in the eyes that mimicked AMD, and then looked at what role macrophages had in the eye abnormalities. They found that in the eyes of mice that lacked IL-10, there was reduced new vessel formation and increased numbers of macrophages compared to mice that had normal amounts of IL-10. Also, preventing macrophages from getting into the eyes of such mice by injecting IL-10 into the eyes made the new vessel formation worse, while direct injection of macrophages made it better.

**What Do These Findings Mean?**

Although animal models cannot completely replicate disease in humans, they can give us an idea of how diseases might come about and suggest possible treatment strategies. It is possible that inhibiting the effect of IL-10, or other strategies that make macrophages more efficient in the eye, may be a useful treatment for AMD. In a related Perspective (DOI: 10.1371/journal.pmed.0030364), Susan Lightman and Virginia Calder discuss the findings further, including suggesting new experiments that will need to be done as a next step.

**Additional Information.**

Please access these Web sites via the online version of this summary at http://dx.doi.org/10.1371/journal.pmed.0030310.

- MedlinePlus encyclopedia entry on macular degeneration
- National Institutes of Health Senior Health page of information on AMD
- National Eye Institute AMD fact page

## Introduction

Inflammation in response to insult and injury can stimulate immunity and promote healing. However, recent work has focused on the role of chronic inflammation in the development of pathologic processes such as cancer, heart disease, and eye disorders. The macrophage component of inflammation has received attention because of macrophage production of proangiogenic and proinflammatory molecules that can fuel disease progression. In cancer, chronic inflammation has been demonstrated within tumor foci, and a number of studies suggest that tumor-associated macrophages promote the growth, proliferation, and metastasis of neoplastic cells as well as stimulate neovascularization, leading to tumor progression [1,2]. Recent research in heart disease has shown that inflammation plays a key role in atherosclerosis. Immune cells have been shown to dominate early atherosclerotic lesions, and molecules produced by these cells accelerate progression of the disease [3].

Recent studies of the blinding eye disorder age-related macular degeneration (AMD) also suggest a role for inflammation, specifically macrophages, in promoting this disease [4,5]. AMD is a progressive disease that causes irreversible visual impairment and blindness in nearly 50 million people globally [6,7]. Current estimates of patients affected with AMD are higher than those of patients affected by Alzheimer disease [8]. Although both geographic atrophy and neovascularization represent advanced forms of AMD, neovascular AMD is the more aggressive form and accounts for almost 90% of blindness from this disease. It is characterized by choroidal neovascularization (CNV), which is the development of abnormal blood vessels underneath the retina. Evidence for a disease-promoting role for macrophages in neovascular AMD derives from two sources. First, studies in a mouse model showed that systemic depletion of macrophages using clodronate-filled liposomes blocked neovascularization [4,5]. Second, certain single nucleotide polymorphisms in complement factor H are observed in a significantly higher percentage of patients with AMD [9–12]. These data, along with the discovery of complement factor H in AMD lesions [10], have been interpreted to suggest that inflammation promotes CNV.

Other recent studies suggest that macrophages may be anti-angiogenic. Mice lacking a macrophage recruitment chemokine (ccl-2) had spontaneous CNV [13], suggesting that when macrophages could not be recruited to the eye, animals were more prone to develop CNV. Another study showed that macrophages are essential to the regression of abnormal vasculature in the anterior segment of the eye [14]. Thus, the role of macrophages in pathogenic neovascularization still remains controversial.

Our study was designed to directly examine the complex role of macrophages in an established mouse model. Although the murine laser model is only a surrogate acute injury model for AMD—which is a chronic disease—it has been remarkably successful in predicting therapies that have since proven to be efficacious in the treatment of AMD [15,16]. It has been also been extremely accurate in revealing the pathophysiology of CNV, the sine qua non of neovascular AMD [4,5,17,18]. Photodynamic therapy, the first treatment for CNV in AMD approved by regulatory agencies in the United States, Europe, Australia, and Japan, was initially examined and proven to be efficacious in an animal model of laser-induced CNV [15,19]. The first human anti-VEGF therapy for CNV in AMD was approved by the United States Food and Drug Administration in 2005. The efficacy of anti-VEGF therapy in ocular neovascularization was initially studied in an animal laser model and was then tested in two large, randomized double-masked human clinical trials that led to the US Food and Drug Administration approval of anti-VEGF therapy for CNV in AMD [16,20]. In a laser-induced mouse model, pigment-epithelium-derived factor was demonstrated to inhibit CNV [21]. The same team of investigators has since completed a phase I trial of adeno-associated virus– pigment-epithelium-derived factor administered intravitreally in patients with CNV from AMD [22]. A preponderance of evidence in the peer-reviewed literature demonstrates that the laser model is a reliable surrogate for understanding the pathophysiology of CNV in AMD. In addition, it has proven to be an excellent model for testing molecules and compounds that successfully treat CNV. The injury to the retinal pigment epithelium (RPE) in AMD is a chronic phenomenon; nevertheless, the laser-mediated injury to the RPE in the mouse model, although acute, might simulate an accelerated phenotype of the chronic disease process. In this paper, we examine the role of innate immunity, specifically macrophage-mediated immunity, in regulating angiogenesis in the eye. We investigate the complex role of macrophages in regulating angiogenesis in an animal model of the human disease AMD and attempt to understand the pathophysiologic mechanisms of CNV that lead to blindness in AMD.

## Methods

### Mouse Strains

C57/BL6 (B6), B6-*IL-10* ^−/−^, B6-*gld,* and B6-*lpr* mice were purchased from Jackson Laboratory (Bar Harbor, Maine, United States). All mouse experiments contained 3–5 mice and were repeated at least three times with similar results. All work was carried out in accordance with Association for Research in Vision and Ophthalmology guidelines (http://www.arvo.org/eweb/dynamicpage.aspx?site=arvo2&webcode=AnimalsResearch).

### Laser-Induced Murine Model of CNV

CNV was induced by rupture of the RPE and underlying Bruch's membrane with a krypton laser in 5- to 7-wk-old mice as described [17,18]. Briefly, four laser spots were placed in each fundus in the peripapillary area using a Krypton Red Laser (614 nm, 50 μm, 0.05 s, 200 mW). After 7 d, the animals were perfused with 3% FITC-conjugated high-molecular-weight dextran (2,000 kDa). A dissecting microscope was used to remove the cornea and lens and gently separate the retina from the underlying choroid and sclera. Microscissors were used to make four radial incisions in the sclero-choroidal eyecup in order to prepare choroidal flat mounts on glass slides. The tissues were incubated in 4% paraformaldehyde for 45 min and washed three times with 3% bovine serum albumin. The tissues were then counter-stained with Cy-3-conjugated anti-mouse elastin antibody for 1 h and washed three times with 3% bovine serum albumin. CNV was identified as FITC-perfused vessels above the plane of Bruch's membrane on confocal microscopy. Images were captured in a three-dimensional stacked fashion for volumetric analysis by Metamorph imaging software (Universal Imaging, Sunnyvale, California, United States). Data were recorded as volume of CNV (microns^3^) ± standard error. CNV volumes for all eyes in a treatment group were averaged and compared individually to controls using Student's *t* test, which has been utilized in similar CNV studies [4,18]. CNV grading was performed in a masked manner, where the individual groups were coded with letters and then presented to the grader for confocal microscopy and quantitative analysis of CNV.

### Cytokine and Antibody Injections

Rat anti-mouse monoclonal antibodies to IL-10 (JES5.2A5) and an isotype-matched (IgG2b) antibody were purchased from Genzyme (Cambridge, Massachusetts, United States). Mice received intravenous injections (500 μg) 1 d prior to laser photocoagulation (day −1) as described previously [23]. Antibody injections were repeated on day 0 (day of laser) and day 1, prior to harvesting of the eyes on day 7 for analysis of CNV. Recombinant mouse IL-10 was purchased from BD Biosciences (San Jose, California, United States). Five microliters (100 ng) was injected into the vitreous cavity of mouse eyes with a 33-gauge needle fitted to a Hamilton syringe on day 0 or 3 after laser treatment. Anti-CD11b (5C6), anti-F4/80 (C1;A3–1), and the isotype control (IgG2b) were purchased from Serotec (Raleigh, North Carolina, United States). We injected 500 μg of each antibody on days −1, 0, and 1. Biotinylated anti–Fas ligand (FasL) (MFL-3) and Strepavidin-FITC for flow cytometry were purchased from BD Biosciences.

### Immunohistochemistry

Sclero-choroidal flat mounts were prepared 7 d after laser treatment. PE-conjugated anti-CD11b antibody (1:100) or isotype-matched control antibody (BD Biosciences) was used to stain the mounts for 1 h at room temperature; the mounts were then washed with PBS three times and analyzed by 3-D confocal microscopy. Numbers of macrophages (CD11b^+^) were counted per lesion (per 20× field centered on the laser lesion), and average numbers were represented. Lesions in flat mounts were also analyzed for neutrophils with PE-conjugated anti-Gr-1 antibody, for dendritic cells with PE-conjugated anti-CD11c, and for T cells with PE-conjugated CD3 antibody (BD Biosciences). PE-conjugated F4/80 antibody (Caltag Laboratories, Burlingame, California, United States) was also used to stain macrophages. PE-conjugated anti-IL-10 antibody (1:100) and isotype control were purchased commercially (BD Biosciences) and used for staining paraffin-sectioned eyes as described above.

### Bone Marrow Culture

Cells for intravitreal injections were prepared from bone marrow culture and spleen. Bone marrow was isolated from proximal limb bones as described previously [24]. Briefly, all muscle tissue was removed from the bones and the bones were washed in 70% alcohol for 5 s prior to two washes in PBS. The ends of the bones were cut with scissors and the marrow harvested by using a syringe and 25-gauge needle to flush the bones with complete RPMI. Then 2 × 10^6^ cells were cultured for 10 d in 10 ml of complete RPMI and 1,000 U/ml GM-CSF in 100-mm petri dishes. On day 3 and 6, an additional 500 U/ml and 1,000 U/ml GM-CSF were added, respectively. On day 10, the non-adherent cells containing dendritic cells were discarded. The adherent cells were mechanically removed and harvested with a cell scraper. CD11b^+^ cells were then isolated by positive selection (see below).

### Positive Selection Using Spleen Cells

CD11b^+^ and F4/80^+^ macrophages were purified from dissociated mouse spleen cells or GM-CSF-cultured macrophages using the PE-positive selection kit (SpinSep, StemCell Technologies, http://www.stemcell.com). CD3^+^ T cells or CD11c^+^ dendritic cells were also isolated by the same protocol. Cell purity was greater than 90% by flow cytometry. Cells were injected into the vitreous cavity of eyes of mice using a 33-gauge Hamilton needle on the same day as laser treatment. Control mice were injected with PBS.

### Transgenic Mice

*VMD2-IL-10* transgenic (Tg) mice were constructed to overexpress IL-10 in RPE cells. VMD2 (Bestrophin) localizes to the basolateral plasma membrane of the RPE [25] (*pVMD2-placF* was provided by Noriko Ezumi, Johns Hopkins Medical School). We removed the *VMD2* promoter and cloned it into the pCI plasmid (Promega, Madison, Wisconsin, United States), replacing the CMV promoter, and then placed the *IL-10* ORF downstream. *VMD2-FN-14* was made by replacing the *IL-10* ORF with the *FN-14* ORF [26]. Tg mice were produced by injecting fertilized mouse oocytes with transgene DNA by standard protocols in the Washington University Department of Ophthalmology and Visual Science Molecular Biology Core facility. Founders were screened by PCR and used for breeding. *IL-10* expression was verified by RT-PCR and immunohistochemistry. Biomicroscopic examination of the anterior and posterior segments of the eye and histopathologic analysis of ocular tissues showed no overt abnormalities. Such mice are viable and have shown normal life spans compared to littermate controls (data not shown).

### Macrophage Activation and Apoptosis Assay

Purified CD11b-F4/80 macrophages were placed into 96-well flat-bottom tissue culture plates (1 × 10^5^ per well). Cells were treated with lipopolysaccharide (LPS) (0.1 μg/ml) or necrotic retinal cells for 3 h at 37 °C and 5% CO_2_ in complete RPMI. L1210-Fas target cells (2 × 10^4^ cells labeled with ^3^H-thymidine) were added and the plates incubated for an additional 16–20 h. Cells were harvested by filtration through glass fiber filters (Packard Instruments, Meriden, Connecticut, United States) using a Filtermate 96-cell harvester (Packard Instruments) and counted on a microplate scintillation counter (Packard Instruments). Data were expressed as percent cell death, calculated as 100 × (counts per minute from L1210-Fas alone – counts per minute of L1210-Fas^+^ macrophages)/counts per minute from L1210-Fas alone.

### Preparation of Necrotic Retinal Cells

Necrotic retinal cells were prepared from B6 mice. Eyes were removed from euthanized mice and dissected in RPMI complete medium. Anterior segment and lens were removed and discarded. The neurosensory retina was gently peeled from the choroid with fine-tip forceps and ground between two glass slides. Cells in each retina were counted and cell number adjusted to 5 × 10^7^ per microliter. The retina was placed in RPMI complete medium and subjected to four freeze/thaw cycles using liquid nitrogen. Ten microliters of this material was added per well of CD11b^+^-F4/80^+^ macrophages.

### Statistics

Student's *t* test was used to perform statistics on all experiments in the study, as described previously [17,18]. Individual numeric values were imported into the statistical package (SigmaPlot for Windows, 2002 version) to compute the *p-*value in order to determine significance. A *p-*value of less than 0.05 was considered to be significant.

## Results

### IL-10 Promotes CNV

CNV in the mouse model is induced by disruption of the RPE and underlying Bruch's membrane with a krypton laser [17,18]. Following laser treatment of the eye, blood vessels penetrate the subretinal space, forming neovascular complexes. The volume of these complexes is indicative of the extent of new vessel growth (i.e., CNV). Values are easily compared among groups in the same experiment. Although only a surrogate for the human disease, the mouse model has been used extensively to study the pathogenesis of experimental neovascularization. The model has also been used in preclinical trials for testing potential therapies for AMD that have since proven effective in preventing vision loss [16,27].

**Figure 1:**
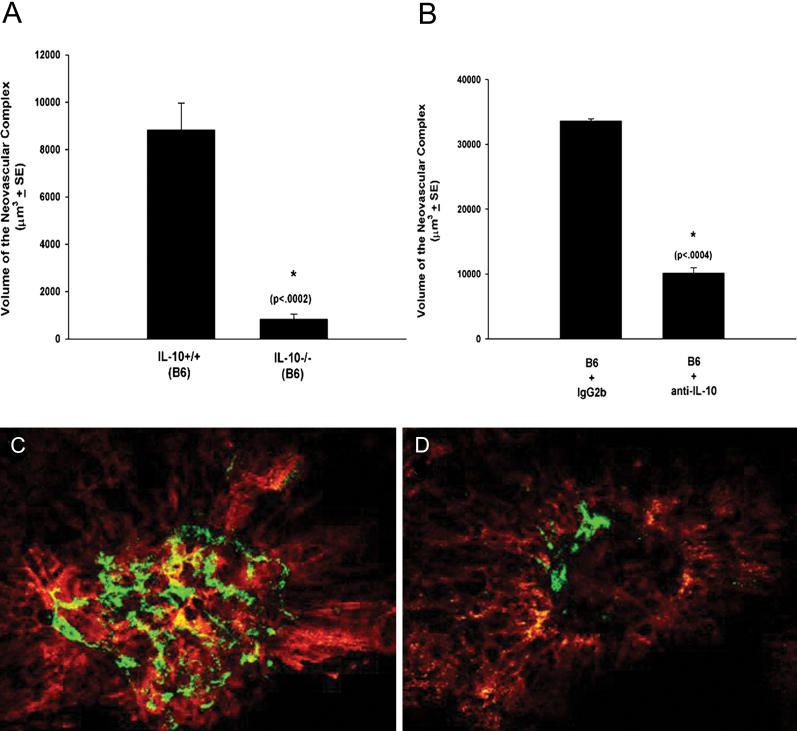
IL-10 and Neovascularization (CNV) (A and B) CNV was induced in *IL-10* ^−/−^ (volume of the neovascular complex: 829.6 ± 225.7 μm^3^) and B6 (*IL-10* ^+/+^; 8,830.7 ± 1,130.3 μm^3^) mice (A) and B6 mice injected on days 0, 3, and 5 with anti-IL-10 (10,115.3 ± 850.5 μm^3^) or isotype control antibody (IgG2b; 33,602.3 ± 317.4 μm^3^) (B). Asterisks indicate values significantly different from control; *p-*values given in parentheses are based on Student's *t* test performed as described [17,18]. (C and D) Seven days following laser treatment, the volumes of the neovascular complexes (green) were determined by confocal microscopy. Shown is representative CNV in a B6 eye (C) and an *IL-10* ^−/−^ eye (D).

We explored the role of inflammation by examining the induction of CNV in *IL-10* ^−/−^ mice. IL-10 is an anti-inflammatory cytokine that inhibits T cell and macrophage functions. It is a potent inhibitor of cytokine and chemokine production, including a number of molecules known to attract monocytes and macrophages to sites of inflammation. The absence of IL-10 typically results in substantial increases in inflammation at multiple sites following diverse stimuli [23,28]. If the current hypothesis is correct, we would expect increased inflammation and increased CNV in *IL-10* ^−/−^ mice. In comparing the volume of the neovascular complexes in *IL-10* ^−/−^ mice and-wild type (wt) B6 mice 7 d following laser treatment, we found that mice lacking IL-10 had significantly decreased new vessel growth (Figure 1A). Systemic neutralization of IL-10 in wt B6 mice also significantly diminished CNV (Figure 1B), recapitulating the effect of the targeted deletion of the *IL-10* gene. Representative lesions from a B6 eye (Figure 1C) and an *IL-10* ^−/−^ eye (Figure 1D) show profound differences in CNV. Since IL-10 is made exclusively by hematopoietic cells [28], it is not surprising that bone-marrow-derived cells were identified as the source of IL-10 in this model (Figure S1).

### IL-10 Inhibits Recruitment of Macrophages to Neovascular Complexes

**Figure 2:**
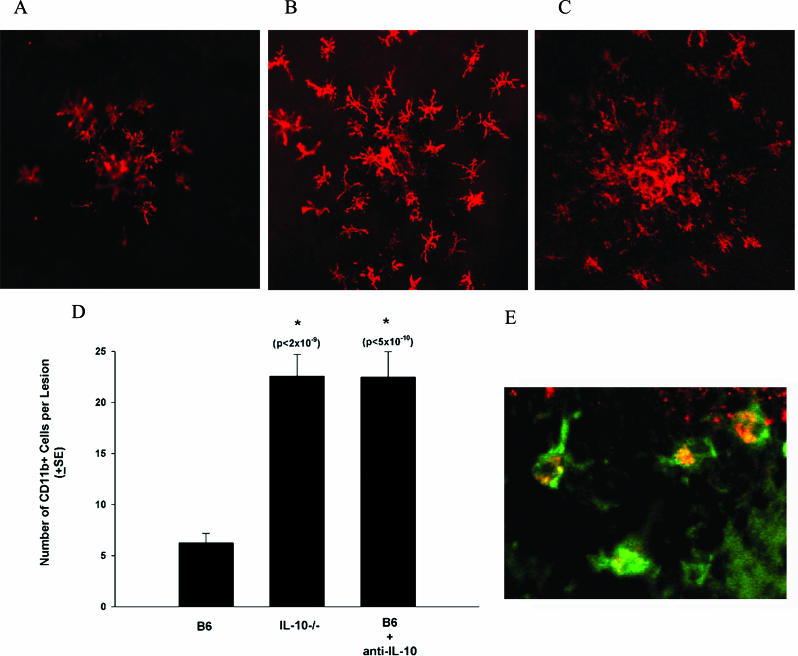
Cellular Infiltrates in CNV Lesions (A–C) On day 7 following laser treatment, whole mount stains were performed to determine the cells present in the area of the neovascular complex. Stains for CD11b were performed on (A) B6, (B) *IL-10* ^−/−^, and (C) B6 mice treated with neutralizing anti-IL-10. Images were take by confocal microscopy (magnification 200×) centered on the laser lesion. (D) The number of CD11b^+^ cells per lesion was counted (200× high-power field centered on the laser lesion) (B6, 6.3 ± 0.9; *IL10* ^−/−^, 22.6 ± 2.1; B6 + anti-IL10, 22.5 ± 2.5). Asterisks indicate values significantly different from control. (E) Dual staining was performed on day 7 following laser treatment using FITC-conjugated anti-CD11b (green) and PE-conjugated anti-F4/80 (red) (magnification 400×).

The effects of IL-10 in this system could be direct, where IL-10 influences endothelial cell function, or they could be the result of the hyperinflammatory response that is characteristic of IL-10-deficient animals. We have been unable to show an effect of IL-10 on vascular endothelial cell proliferation or tube formation in vitro (data not shown), and we have been unsuccessful in identifying IL-10 receptors on vascular endothelial cells. Consequently, we tested whether our observations in *IL-10* ^−/−^ mice and wt mice receiving anti-IL-10 were related to the increased inflammatory phenotype observed in these animals. Examination of the neovascular complexes in wt mice (Figure 2A), *IL-10* ^−/−^ mice (Figure 2B), and wt mice that were treated with neutralizing anti-IL-10 (Figure 2C) revealed significantly more CD11b^+^ cells in *IL-10* ^−/−^ mice and wt mice treated with neutralizing anti-IL-10 than in wt controls. Quantitation of CD11b^+^ cells in CNV lesions on day 7 confirmed that there are substantial increases in the number of these cells when IL-10 is not present (Figure 2D). Immunohistochemical stains for other CD11b^+^ cells such as dendritic cells (CD11c) and neutrophils (Gr-1) were negative, as were stains for T cells (CD3) (data not shown). Immunostaining for CD11b and F4/80 (Figure 2E) confirmed that these infiltrating cells were macrophages. While these data are consistent with the hyperinflammatory state observed in the absence of IL-10, they are not consistent with the idea that macrophages promote CNV. Since mice with low amounts of CNV had increased infiltration of CD11b^+^-F4/80^+^ inflammatory cells and a paucity of other immune cells, a more likely explanation is that CD11b^+^-F4/80^+^ macrophages are either inhibiting blood vessel formation in the retina or promoting vascular endothelial cell death.

### Inhibiting Macrophage Recruitment Promotes CNV

**Figure 3:**
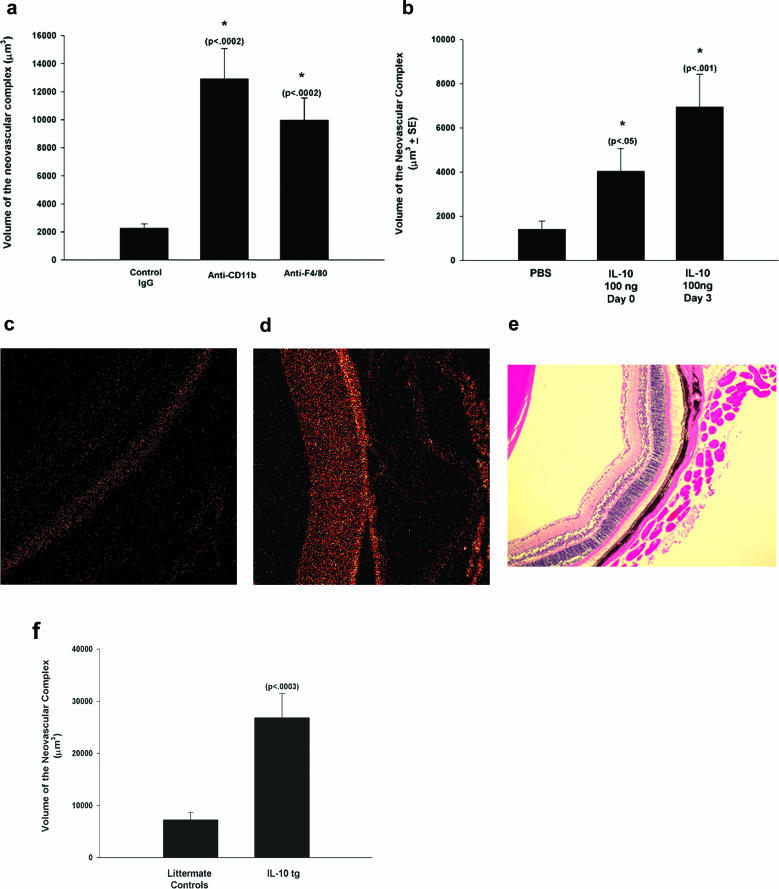
Inhibition of Macrophage Infiltration Promotes CNV (A) *IL-10* ^−/−^ mice were injected with anti-CD11b (volume of the neovascular complex: 12,903.1 ± 2,171.3 μm^3^), anti-F4/80 (9,977.3 ± 1,572.7 μm^3^), or control IgG (2,262.3 ± 313.4 μm^3^) on days −1, 0, and +1. CNV volumes were determined on day 7. (B) *IL-10* ^−/−^ mice were injected in the vitreous with PBS (1,394 ± 382.4 μm^3^) or 100 ng of *rIL-10* on day 0 (the day of laser treatment; 4,033.6 ± 1,026.5 μm^3^) or day 3 (6,949.8 ± 1,475.5 μm^3^). (C and D) Cross-sections of the retinas of a control littermate (C) and a *VMD2-IL-10* Tg mouse (D) stained with anti-IL-10 and examined by confocal microscopy. (E) Hematoxylin and eosin staining of the retina of a *VMD2-IL-10* Tg mouse (magnification 200×). (F) *VMD2-IL-10* Tg mice (24,428.6 ± 4,360.7 μm^3^) and littermate controls (6,830.1 ± 1,324.9 μm^3^) were subjected to laser treatment and CNV volumes were determined on day 7. Asterisks indicate values significantly different from control; *p-*values are given in parentheses.

We directly tested this idea with three types of experiments. First, we used systemic injection of anti-CD11b or anti-F4/80, treatments known to prevent entry of macrophages into tissue [29]. Figure 3A shows that depletion of CD11b^+^ cells with anti-CD11b as well as depletion of macrophages with anti-F4/80 led to significantly increased neovascularization. Examination of the lesions in treated mice revealed that both treatments abolished the migration of macrophages into the laser lesions (data not shown). This finding further substantiates that the presence of macrophages inhibits angiogenesis in the retina.

Next, the effect of intraocular IL-10 on CNV was explored by administering IL-10 (100 ng) directly into the eye of *IL-10* ^−/−^ mice. This resulted in a significant increase in neovascularization that approached control levels (Figure 3B), with injection of IL-10 on day 3 being more effective than injection on day 0 (day of laser treatment). Thus, injection of IL-10, which prevents inflammatory cell recruitment to sites of inflammation [23,28], increased the level of CNV.

Based on these findings, we hypothesized that high levels of IL-10 would prevent macrophage entry into the eye, resulting in increased pathologic CNV. To test this idea, we developed a Tg mouse overexpressing *IL-10* in the RPE using the human *VMD2* promoter. These *IL-10* Tg mice (or *VMD2-IL-10*) expressed high levels of secreted IL-10 in the retina (Figure 3D) compared to transgene-negative mice (Figure 3C), but had completely normal retinal architecture (Figure 3E). When CNV was induced by laser treatment (Figure 3F), neovascularization was substantially elevated over that in controls. In addition, *VMD2-IL-10* Tg mice did not show macrophage infiltrates in the CNV lesions (data not shown). Thus, direct injection of IL-10 and Tg overexpression of *IL-10* both augmented new vessel formation. We conclude from these experiments (Figures 2 and 3) that inhibition of macrophage recruitment leads to increased levels of CNV.

### Macrophages Directly Inhibit CNV

**Figure 4:**
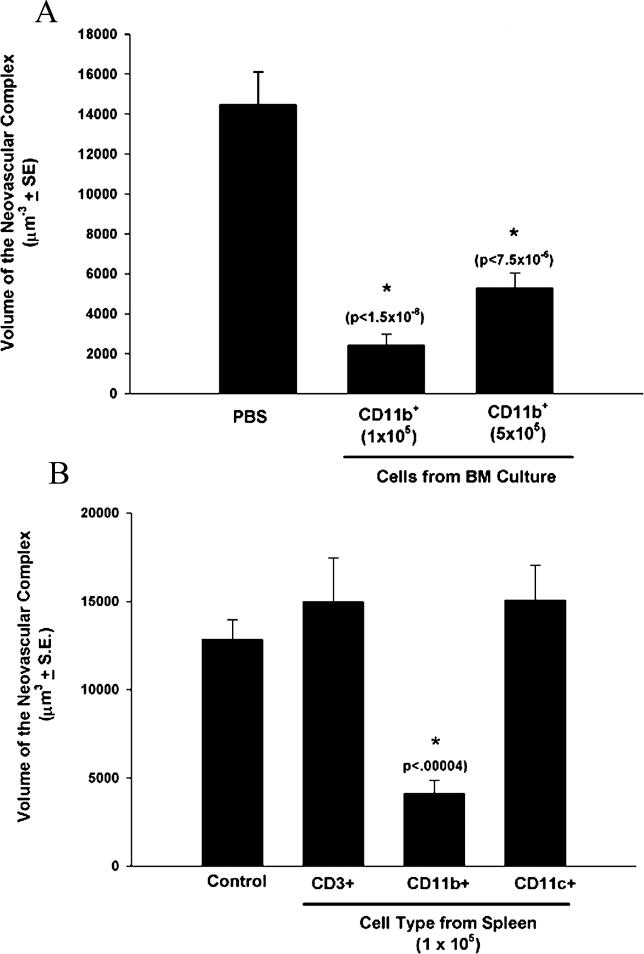
Injection of Macrophages Inhibits CNV Cells obtained from bone marrow (BM) cultures (A) or spleen (B) were purified by magnetic beads and injected into the vitreous cavity on the day of laser treatment. Seven days later, the volume of the neovascular complex was determined by confocal microscopy. Asterisks indicate values significantly different from control; *p-*values given in parentheses are based on Student's *t* test. (A) Volume of the neovascular complex for injection of PBS (14,470.9 ± 1,636.4 μm^3^), CD11b cells—1 × 10^5^ cells (2,431.2 ± 551.3 μm^3^), and CD11b cells—5 × 10^5^ cells (5,274.2 ± 772.5 μm^3^). (B) Volume of the neovascular complex for injection of PBS (12,857.9 ± 1,094.9 μm^3^), CD3 cells (14,960.5 ± 2,502.0 μm^3^), CD11b cells (4,135.1 ± 714.4 μm^3^), and CD11c cells (15,063.3 ± 1,982.4 μm^3^).

We directly tested whether macrophages inhibited CNV by injecting cells into the vitreous cavity at the time of laser treatment. Figure 4A shows that injection of CD11b^+^ cells obtained from bone marrow cultures significantly reduced CNV. Inhibition was observed with injection of 1 × 10^5^ and 5 × 10^5^ cells. CD11b^+^ cells obtained from spleen by positive selection (>90% F4/80^+^; data not shown) also significantly inhibited CNV (Figure 4B), while splenic CD11c^+^ (dendritic) and CD3^+^ (T cells) were not effective. These data further support the idea that macrophages inhibit, rather than promote, CNV. The effect is attributable to macrophages and not dendritic cells as CD11c^+^ dendritic cells and T cells failed to inhibit CNV.

### Macrophages Inhibit CNV via FasL-Mediated Cell Death

**Figure 5:**
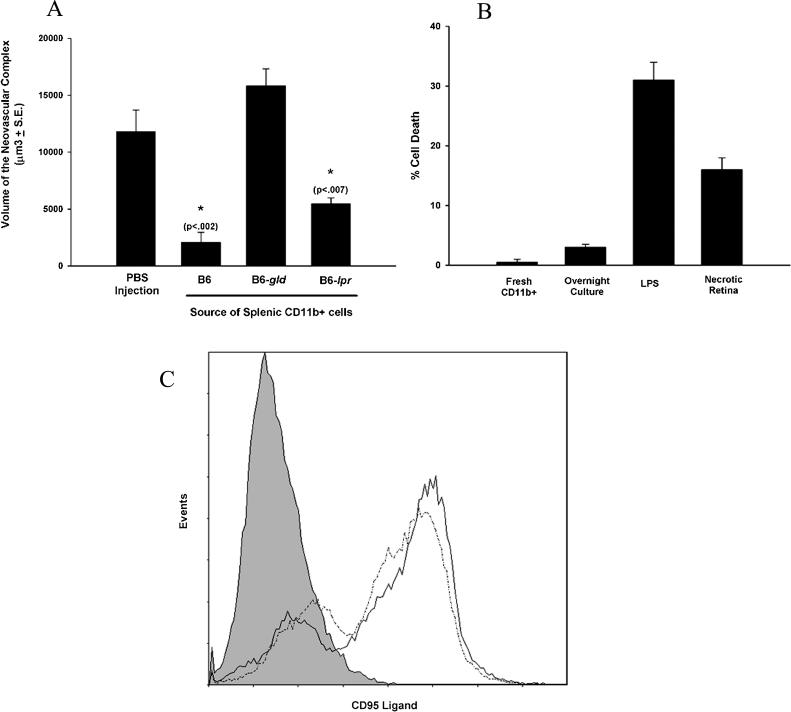
Macrophages Inhibit CNV via CD95L (A) CD11b^+^ cells from B6, B6-*lpr,* and B6-*gld* mice were purified from spleen, and 1 × 10^5^ cells were injected into the vitreous cavity on the day of laser induction of CNV. Seven days later, the volume of the neovascular complex was determined by confocal microscopy for PBS (11,786.4 ± 1,907.3 μm^3^), B6 (2,084.6 ± 874.8 μm^3^), *gld* (15,824.9 ± 1,483.6 μm^3^), and *lpr* (5,443.9 ± 542.1 μm^3^). Asterisks indicate values significantly different from control. (B) Purified CD11b^+^ cells were cultured overnight with LPS or necrotic retina and tested for killing against L1210-Fas. (C) The expression of CD95L in CD11b cells was determined by flow cytometry following overnight culture with LPS (dotted line) or necrotic retina (solid line). The line with shading underneath represents untreated cells.

Inhibition of neovascularization by macrophages could be mediated by a direct effect of the cells on the developing vessels (via contact or through paracrine mechanisms), or it could be the result of a secondary effect of these cells on blood vessel formation through other resident or infiltrating cells. CD95/CD95L interactions are known to regulate vessel growth in the eye in the current model [17]. This regulation operates through the interaction of CD95L with the Fas antigen (CD95) on the choroidal blood vessels induced to grow by laser treatment. The CD95/CD95L pathway was examined by injecting purified CD11b^+^ cells from *lpr* (CD95-deficient), *gld* (CD95L-deficient), or wt (B6) mice into the vitreous to assess the effect on vessel formation. The results in Figure 5A show that CD11b^+^ cells lacking functional CD95L *(gld)* did not inhibit CNV. Thus, CD95L derived from CD11b^+^ cells is responsible for inhibiting new vessel formation. This effect is not due to the inability of *gld* macrophages to home to the laser lesion after injection since there was no difference in the number of carboxyfluorescein diacetate succinimidyl ester (CFSE)–labeled wt or *gld* macrophages per lesion when they were examined 3 d after injection (Figure S2).

Macrophages are known to both promote and inhibit inflammatory responses, but they are not typically FasL^+^ unless stimulated [30]. This suggests that activation (or at least interaction) with the damaged retinal tissue might promote CD95L expression, leading to acquisition of killing function. CD11b^+^ cells purified from spleen were subjected to necrotic cells (from the retina) or LPS and then tested for their ability to kill CD95^+^ targets. As shown in Figure 5B, cells fed necrotic cells or treated with LPS were able to kill CD95^+^ targets. This was through upregulation of CD95L on macrophages by both stimuli (Figure 5C).

## Discussion

Our data show that macrophages are protective against the development of CNV in the eye and that IL-10 regulates macrophage function. This finding suggests that in AMD a functioning immune system is important in preventing blindness from this disease. Since current treatments limit vision loss but are not specifically designed to prevent or reverse CNV, our results also suggest a novel approach to the design of therapies.

Inflammation, specifically macrophage infiltration, is known to promote a number of pathologic processes including cancer, heart disease, and eye disorders. The ability of these cells to produce a variety of molecules that stimulate blood vessel growth is thought to be an important component in disease progression. However, macrophage function is highly complex as these cells can also promote wound healing [31,32] and control vessel growth during developmental processes [14]. Our results demonstrate a potentially new role for macrophages in blinding eye disorders, where macrophages may not be proangiogenic but function to limit the spread of choroidal blood vessels in the eye. This effect on CNV has implications for neovascular AMD, where CNV is the major pathogenic factor.

Advanced AMD that leads to severe visual loss affects almost 2 million people in the United States [33]. By 2020, the number of patients with all stages of AMD in the industrialized world is expected to increase by about 50%. AMD now accounts for over 40% of blindness among the institutionalized elderly [33,34]. Patients with advanced AMD rate their quality of life lower than patients with AIDS and dialysis-dependent renal failure [35,36]. Current treatments are designed to limit further visual loss, but these are only marginally effective [16]. There is no treatment that can reverse the damage to vision. Consequently, insights into the etiology of CNV associated with AMD, as described in this report, add significantly to the information needed to design effective treatments.

While it is clear that the immune system can play a role in neovascularization, there has been conflicting evidence regarding the role of the immune system in neovascular AMD. The most popular idea is that macrophages (and inflammation) are proangiogenic. Studies suggesting this used the laser model described here and systemically depleted phagocytic cells by injection of the compound clodronate encapsulated in liposomes [4,5]. Since systemic depletion of phagocytes decreased the level of CNV, the authors concluded that macrophages promote CNV. When we examined this system, we found that liposomes can actually enter developing endothelial cells found in the laser-induced neovascular complexes (Figure S3). These findings, coupled with the fact that liposomes are known to enter a wide variety of other cells [37,38], suggest that the effects observed may be due to the toxicity of the clodronate liposomes directly on sprouting endothelial cells.

A recent study suggested that macrophages may be anti-angiogenic. These authors demonstrated that mice lacking a macrophage recruitment chemokine (ccl-2) had spontaneous CNV [13]. Although these authors did not discuss the possibility, this observation suggests that the inability to recruit macrophages into the eye leads to the development of CNV. Not only do our results support this idea, but we have directly tested it in the laser model of CNV. Our study shows that increased macrophage influx inhibits CNV, while treatments that prevent macrophage entry into the eye suppress the anti-angiogenic response of macrophages. Data from our study complement the natural history of AMD and the etiology of inflammatory eye diseases in humans. Although leukocytes including macrophages, lymphocytes, erythrocytes, and other cells of hematopoietic origin have been isolated from samples of CNV and identified by immunohistochemistry or electron microscopy, there is no conclusive evidence about whether these cells are pro- or anti-angiogenic [39–41]. The possibility remains that these cells represent an epiphenomenon related to the growth of abnormal blood vessels in the subretinal space. Our data demonstrate a novel role for macrophages in the CNV lesions that is of potential therapeutic relevance. Clinical observations show that CNV in uveitis patients, who have significant inflammation in the eye, is less aggressive [42]. Our findings also complement recent evidence that macrophages are anti-angiogenic during development [14] and in tumors [2]. Based on this information and the data presented in this report we conclude that inflammation may be protective.

Recent genetic-linkage-based evidence suggests the potential importance of complement factor H in AMD [9,11,12]. However, the lack of functional data leaves these important observations still open to interpretation, as they are also consistent with the idea that a healthy immune system (e.g., complement pathway) may actually protect against blindness. We would suggest that one explanation for the linkage between complement factor H and AMD might be that polymorphisms that lead to increased AMD are associated with reduced macrophage recruitment to the chorio-retinal tissues. The elderly, who are at risk for the development of AMD, are known to have compromised immune function [43]. Thus, a disabled macrophage response may be an additional risk factor for the development of CNV and blindness. We would further suggest that physiologic immune surveillance and normal macrophage function are critical in controlling pathologic neovascularization.

The pivotal role for IL-10 in this model is likely related to the anti-inflammatory properties of the cytokine and its ability to inhibit cytokines that attract macrophages [23,28]. We can find no effect of IL-10 on endothelial cells (data not shown). Increases in chemotactic cytokines would be predicted when IL-10 is not present, and one would expect that the loss of cytokines that promote infiltration of monocytes or macrophages would predispose to AMD.

Our studies also demonstrate that macrophages use FasL to inhibit CNV. We have demonstrated previously that FasL expressed on RPE cells is important for the control of CNV [17]: mice defective in the expression of FasL on RPE cells had massive increases in CNV. We believe that our current observations complement this finding, since we believe that FasL on both RPE cells and macrophages is necessary. The loss of FasL on RPE cells leads to substantial (5- to 8-fold) increases in CNV [17], and we would not expect the few macrophages that enter the lesion (10–20 per lesion) to be able to control this high level of CNV even if they are FasL^+^. When FasL is present on the RPE, as occurs in most experiments, the CNV is more modest and then amenable to control by the FasL^+^ macrophages that infiltrate the lesions. It should be noted that the role of macrophage-expressed FasL was revealed only when we injected large numbers of FasL^+^ macrophage into the vitreous (1–5 × 10^5^). Therefore, we conclude that RPE-derived FasL and macrophage-derived FasL are important in controlling CNV.

Our data suggest potential sites for therapeutic intervention in AMD. Since the absence of IL-10 results in increased macrophage influx and reduced neovascularization, novel targeting therapies designed to inhibit local IL-10 function, promote tissue-specific macrophage activity, and/or target the CD95/CD95L pathway should be considered as treatments for neovascular AMD. IL-10 activity in the eye can be inhibited by local administration of neutralizing antibodies, inhibitory RNA, or aptamers. Although the most immediate application for this form of therapy would presumably be in patients with preexisting CNV in order to limit or reverse vision loss, long-term inhibition of IL-10 function in the eye could potentially be used as preventive therapy in patients with dry AMD who are at high risk for developing CNV. In addition to demonstrating that IL-10 is an attractive therapeutic target in AMD, we have also shown that the role of macrophages in angiogenesis might be more complex than previously suggested. The anti-angiogenic functions of macrophages should be considered in evaluating any therapies that might nonselectively neutralize all macrophage effector function because of the potentially devastating consequences on the disease process and vision in patients with AMD.

## Supporting Information

Figure S1 Source of IL-10 Bone marrow chimeras were generated to test the source IL-10. *IL-10* ^−/−^ mice that were reconstituted with B6 bone marrow (BM) (volume of the neovascular complex: 10,143.9 ± 1,962.8 μm^3^) showed levels of CNV comparable to B6 mice (12,756.7 ± 3,744.4 μm^3^). In contrast, B6 mice that were reconstituted with *IL-10* ^−/−^ bone marrow (3,292.7 ± 632.9 μm^3^) showed levels of CNV similar to *IL-10* ^−/−^ mice (3,758.7 ± 1,259.3 μm^3^). This finding demonstrates the source of IL-10 is hematopoietic cells. Asterisks indicate values significantly different from control.
(445 KB TIF)

Figure S2 Homing of CFSE-Labeled Macrophages to CNV Lesions Macrophages from B6-wt or B6-*gld* mice were labeled with CFSE and injected into the vitreous cavity on the day of laser treatment. The number of macrophages per laser lesion were counted on day 3 and were not significantly different for B6 and *gld* mice (9.5 ± 1.4 and 7.6 ± 2.0, respectively; *p* = 0.24).
(593 KB TIF)

Figure S3 Green-Fluorescent-Protein-Labeled Liposomes in CNV Lesions Green-fluorescent-protein-labeled liposomes were injected intravenously into B6-wt mice on the day of laser treatment. The presence of liposomes in the developing blood vessels within the laser lesions was analyzed on day 3. Shown is a representative lesion that demonstrates detectable liposomes in the laser lesion.
(234 KB TIF)

Protocol S1 Additional Methods (60 KB DOC)

### Accession Numbers

The NCBI accession numbers for the mouse genes and gene products discussed in this paper are *Fas* gene (NM_007987), Fas protein (NP_032013), *FasL* gene (NM_010177), FasL protein (AAB33780), *IL-10* gene (NC_000067), and IL-10 protein (NP_034678).

## Abbreviations

AMD: age-related macular degeneration
B6: C57/BL6
CFSE: carboxyfluorescein diacetate succinimidyl ester
CNV: choroidal neovascularization
FasL: Fas ligand
LPS: lipopolysaccharide
RPE: retinal pigment epithelium
Tg: transgenic
wt: wild-type
