# Supplementary material for: Macrophages Inhibit Neovascularization in a Murine Model of Age-Related Macular Degeneration
Source: PLoS Med. 2006 Aug 15;3(8):e310. doi: 10.1371/journal.pmed.0030310 (PMC1539093; doi:10.1371/journal.pmed.0030310)
Supplement: Supplementary file 1 [file pmed.0030310.sd001.doc]

, 711-20 (1998).

**Supporting Methods**

Liposome Preparation and Injection. Liposomes were prepared as described previously in the literature (Van Rooijen 1989; Espinosa-Heidmann et al. 2003). Briefly, 75 mg phosphatidylcholine (Sigma Inc., St. Louis) and 11 mg Cholesterol (Sigma Inc., St. Louis) were dissolved in 20 mL methanol/cholorform (1:1) at 37ºC on a stirrer. The organic phase was removed with vacuum over 2 hours. GFP (BD Biosciences (100 μg in 100 μL -1mg/mL) was dissolved in 10 mL PBS and added to the liposomes over a shaker. The mixture was incubated for 2 hours at room temperature and sonicated in a 37ºC water bath for 3 minutes. The liposomes were then incubated at room temperature for 2 hours and centrifuged at 100000 x g (45,000 RPM) for 30 minutes at 16º C. The supernatant was removed and the liposomes were resuspended in PBS. GFP labeling of liposomes was confirmed by fluorescent microscopy. Mice were injected with liposomes intravenously on days -2, 0 (day of laser), and day 2. Eyes were harvested for imaging at day 3.

CFSE labeling of macrophages. CFSE [1 μL of 5mM CFSE] was added to 1 X 10^6^ purified macrophages (1:1000 dilution) from either C57BL6 -wt or *gld* mice. Cells were incubated 10 minutes at 37ºC in a water bath and washed three times. Labeling was confirmed by fluorescent microscopy.

Bone Marrow Chimeras. Bone marrow chimeras are generated as described previously (Bonfoco et al. 1998). Host mice receive 1100 R of external beam radiation in order to deplete bone marrow cells. Syngeneic donor mice are euthanized and bone marrow cells are harvested from the proximal limb bones. Host mice receive 1 X 10^8^ bone marrow cells intravenously. The mice are observed for three weeks in order to allow the donor cells to replenish the host marrow and form true chimeras prior to assessing the CNV response. Bone marrow cells in the host animal are now derived from and function like donor marrow cells.

**Supporting Figure Legends**

**FIGURE S1**

Source of IL-10. Bone marrow chimeras were generated to test the source IL-10. IL-10 -/- mice that were reconstituted with B6 bone marrow (BM)(10143.9±1962.8) showed control levels of CNV comparable to B6 mice (12756.7±3744.4). In contrast, B6 mice that were reconstituted with IL-10-/- bone marrow (3292.7±632.9) showed levels of CNV similar to IL-10-/- mice (3758.7±1259.3). This data demonstrates the source of IL-10 is from hematopoietic cells. *significantly different from control.

**FIGURE S2**

Homing of CFSE-labeled macrophages to CNV lesions. Macrophages from B6-wt or B6-*gld* mice were labeled with CFSE and injected in to the vitreous cavity on the day of laser treatment. The number of macrophages per laser lesion [B6 (9.5±1.4), gld (7.6±2.0)]were counted on day 3. (p = 0.24).

**FIGURE S3**

GFP-labeled liposomes in CNV lesions. GFP-labeled liposomes were injected intravenously in to B6-wt mice on the day of laser. Presence of liposomes in the developing blood vessels within the laser lesions was analyzed on day 3. The figure is a representative lesion that demonstrates detectable liposomes in the laser lesion.

**References**

Bonfoco E, Stuart PM, Brunner T, Lin T, Griffith TS et al. (1998) Inducible nonlymphoid expression of Fas ligand is responsible for superantigen-induced peripheral deletion of T cells. Immunity 9(5): 711-720.

Espinosa-Heidmann DG, Suner IJ, Hernandez EP, Monroy D, Csaky KG et al. (2003) Macrophage depletion diminishes lesion size and severity in experimental choroidal neovascularization. Invest Ophthalmol Vis Sci 44(8): 3586-3592.

Van Rooijen N (1989) The liposome-mediated macrophage 'suicide' technique. J Immunol Methods 124(1): 1-6.
